# Multi-omics analysis reveals the modulatory effects of *Moringa oleifera* ethanol extract on gut microbiota and metabolic profiles in broilers

**DOI:** 10.3389/fmicb.2026.1899071

**Published:** 2026-08-19

**Authors:** Fuhong Lei, Chunpan Zhao, Fei Wang, Leng Wang, Jiming Long, Zhiliang Ma, Haiquan Li, Zubing Zhang

**Affiliations:** Yunnan Institute of Tropical Crops, Xishuangbanna, China

**Keywords:** bacitracin zinc, broilers, gut microbiota, microbial metabolomics, *Moringa oleifera* ethanolic extract

## Abstract

**Background:**

The overuse of antimicrobial growth promoters (AGPs) in poultry farming has raised global concerns about antimicrobial resistance and food safety, prompting the search for effective natural alternatives. Moringa is rich in bioactive compounds with antimicrobial and immunomodulatory properties; however, its effects on the cecal microbiota and metabolome of broiler chickens when administered via drinking water remain unclear.

**Methods:**

This study employed 16S rDNA sequencing and non-targeted metabolomics to evaluate the effects of *Moringa oleifera* ethanol extract (MOE) and the broad-spectrum antibiotic bacitracin zinc (BZ) on the cecal microbiota, metabolite profiles, and their correlations in broiler chickens.

**Results:**

The findings demonstrated MOE did not significantly affect α-diversity but altered β-diversity, resulting in a clear separation from the control and BZ groups. Linear discriminant analysis effect size (LEfSe) analysis identified Lachnospiraceae and Methanobacteriaceae as key differential biomarkers in the MOE group, whereas Deferribacterales was selectively enriched in the BZ group. At the phylum level, MOE enriched the Euryarchaeota, while BZ increased the abundance of potentially pathogenic phyla, including the Desulfobacterota and Proteobacteria. At the genus level, MOE promoted the growth of beneficial genera such as *Faecalibacterium, Lachnoclostridium*, and *Alistipes*, whereas BZ exhibited a bidirectional effect, increasing both pathogenic and beneficial groups. Metabolomic analysis revealed that MOE significantly regulated the metabolism of sphingolipids, tryptophan, and polyamines, as well as that of vitamin D3 and nicotinamide, whereas BZ primarily activated lipid metabolism and inhibited arginine-related antioxidant pathways. Correlation analysis further established genus-metabolite networks centered on *Lachnoclostridium* and *Lactobacillus* in the MOE group, and *Alistipes* and *Collinsella* in the BZ group.

**Conclusion:**

Overall, MOE is associated with alterations in cecal microbiota and host metabolism by enriching butyrate-producing bacteria and methanogenic archaea, accompanied by extensive metabolic reprogramming involving immune-related and antioxidant pathways.

## 1 Introduction

The poultry industry has emerged as a significant global supplier of animal protein (Birhanu et al., 2023; Castro et al., 2023), with its production growth heavily reliant on antibiotic growth promoters (AGPs) to improve production efficiency and manage disease outbreaks. However, the rising incidence of multidrug-resistant pathogenic bacteria and the detection of antibiotic residues in poultry products have intensified public opposition to the inclusion of AGPs in the feed of food-producing animals (Ma et al., 2021). Consequently, there is an urgent need to formulate a globally coordinated strategy to address the sharp rise in antimicrobial resistance. In this context, plant extracts and their bioactive compounds, such as flavonoids, have garnered considerable attention as potential alternatives to antibiotics (Liu et al., 2026). These natural substances are highly versatile, leaving no toxic residues in animal products (Long et al., 2020), while also regulating intestinal function, promoting nutrient utilization, and enhancing resistance to intestinal stress (Natsir et al., 2024). Consequently, plant-derived additives rich in active metabolites are regarded as viable candidates for replacing AGPs in poultry feed (Utami et al., 2023). Previous studies have explored the nutritional and health-promoting potential of several plants in poultry feed, including neem, papaya, bamboo (Oloruntola et al., 2019) and turmeric (Ogbuewu et al., 2022). Among these, moringa stands out due to its exceptional nutritional profile and rich phytochemical composition.

*Moringa oleifera* Lam. (Moringa), which belongs to the *Moringaceae* family, is commonly referred to as the “miracle tree” (Afrianto and Metananda, 2023; Panova et al., 2025) and has a wide range of uses (Pop et al., 2022); its various parts, including leaves, seeds, roots, bark, fruits, pods, oil, and flowers, offer significant environmental and health benefits (Pareek et al., 2023). Moringa is abundant in bioactive compounds (Husien et al., 2024) and possesses exceptional nutritional and phytochemical value (Pareek et al., 2023), having become an important plant for nutritional health and medicinal purposes (Husien et al., 2024). The leaves and seeds, or their extracts, are particularly rich in proteins, carbohydrates and minerals such as calcium, phosphorus, iron and zinc, as well as vitamins including B-complex, C and E (Abd El-Hack et al., 2022; Liang et al., 2019). They also contain beneficial bioactive compounds with antibacterial and antioxidant properties, including flavonoids, quercetin, catechins, alkaloids, kaempferol, chlorogenic acid, and rutin (Abdalla et al., 2022; Prabakaran et al., 2018; Hamid et al., 2025). Research indicates that these active components can counteract the detrimental effects of free radicals, reduce inflammation (Vergara-Jimenez et al., 2017), promote rapid growth rates in poultry, improve gut health and immune function (Komurcu et al., 2026; Husien et al., 2025), enhance the nutritional quality of poultry products, and inhibit the proliferation of pathogenic microorganisms in the gut (Egbu et al., 2024). Furthermore, moringa exhibits significant antibacterial and antifungal properties, indicating its potential as a natural growth promoter in poultry (Fahey, 2005). Consequently, this study involved the addition of MOE to the drinking water of broiler chickens, with a blank control group and an antibiotic group serving as controls. The aim was to investigate its effects on the microbiota of the broiler cecum and their metabolites, and to evaluate the feasibility of using MOE as a natural alternative to antibiotics in broiler chickens, with a view to providing a scientific basis for the development and utilization of MOE as a feed additive.

## 2 Materials and methods

The animal experiments in this study were carried out at the Yunnan Institute of Tropical Crops.

### 2.1 Experimental design and dietary treatments

A total of 144, 14-day-old Chahua chickens were selected and randomly divided into three groups of 48 chickens each. Each group was then divided into six replicates of eight chickens. The experimental feed primarily consisted of corn and soybean meal. The control group (CK) received a basal diet along with fresh drinking water, while the antibiotic group (BZ) was provided with a basal diet and drinking water supplemented with 100 mg/L of bacitracin zinc. The MOE groups received 30.0 mL/L *Moringa oleifera* ethanolic extract in their drinking water: the basal diet formulation was prepared as a powdered compound feed based on the nutrient requirements for poultry as outlined by the National Research Council (NRC) (1994), as detailed in Table 1.

**Table 1:** Composition and nutrient levels of the basal diet (air-dry basis, %).

Items	Content (%)
Ingredient (%)	
Corn	57.45
Soybean meal	25.87
Fish meal	7.00
Soybean oil	3.70
*L*-Lys·H_2_SO_4_ (98.5%)	0.10
*DL*-Met (99%)	0.10
Limestone	0.50
CaHPO_4_	1.08
Nacl	0.20
Premix	4.00
Nutrient levels	
ME/(MJ/kg)	12.54
CP (%)	19.00
Lys (%)	1.25
Met (%)	0.46
Ca (%)	0.90
P (%)	0.47

Premix provided the following per kilogram of diet: Vitamin A: 10,000 IU; Vitamin D3: 5,000 IU; Vitamin E: 20.0 mg; Vitamin K3: 2.5 mg; Vitamin B1: 2.0 mg; Riboflavin: 8 mg; Vitamin B6: 3.5 mg; Vitamin B12: 0.08 mg; Pantothenic acid: 10.0 mg; Nicotinic acid: 35.0 mg; Biotin: 0.2 mg; Folic acid: 1.0 mg. Manganese (MnSO4·H_2_O): 100 mg; Zinc (ZnSO4·7H_2_O): 80 mg; Iron (FeSO4·7H_2_O): 80 mg; Copper (CuSO4·5H_2_O): 9 mg; Iodine (KI): 0.35 mg; Selenium (Na_2_SeO3), 0.15 mg.

### 2.2 Sample collection and analytical methods

#### 2.2.1 Cecal microbiota analysis

The chickens were slaughtered at 56 days of age, six cecal chyme were collected from each group of chickens. The abdominal cavities of the chickens were quickly opened, and the outer walls of the ceca were disinfected with 75% ethanol. Approximately 1 gram of the cecal contents was taken and placed in a sterilized cryotube, which was temporarily stored in liquid nitrogen. After the sampling was completed, it was transferred to a −80 °C ultra-low temperature refrigerator for future use and sent to Beijing Nuohe Zhiyuan Technology Co., Ltd. (China) for 16S rDNA sequencing. The upstream primer 341F (5′-CCTAYGGGRBGCASCAG3′) and the downstream primer 806R (5′-GGACTACNNGGGTATCTAAT3′) were used to perform PCR amplification of the V3–V4 variable region of the 16S rRNA gene. The gene sequences were analyzed using Qiime1, including α diversity, β diversity, phylum, and class composition.

#### 2.2.2 Cecal metabolomics analysis

Precisely measure 100 mg of caecal contents and thoroughly grind them using liquid nitrogen until a fine powder is obtained. Transfer the pulverized sample into a 2 mL centrifuge tube. Add 1 mL of pre-chilled (−20 °C) 80% methanol solution and vortex for 30 s to ensure complete resuspension of the sample. Incubate the mixture on ice for 5 mins, followed by centrifugation at 4 °C and 15,000 g for 20 mins. Isolate a portion of the supernatant and dilute it to achieve a final methanol concentration of 53% for analysis using LC-MS-grade water. Transfer the diluted sample to a new Eppendorf tube and centrifuge again at 4 °C and 15,000 g for 20 mins. Subsequently, inject the supernatant into an LC-MS/MS system for analysis. The UHPLC-MS/MS analysis was conducted at Novogene Co., Ltd. (Beijing, China), utilizing a Vanquish ultra-high-performance liquid chromatography system (Thermo Fisher, Germany), in conjunction with an Orbitrap Q Exactive™ HF-X mass spectrometer (Thermo Fisher, Germany). Samples were injected at a flow rate of 0.2 mL/min using a 12-min linear gradient on a Hypersil Gold column (100 mm × 2.1 mm, 1.9 μm). The eluents employed for positive and negative ion modes were Eluent A (0.1% formic acid in water) and Eluent B (methanol), respectively. The solvent gradient was configured as follows: 2% B for 1.5 mins; a linear increase from 2% to 85% B over 3 mins; 85% to 100% B over 10 mins; a decrease from 100% to 2% B over 10.1 mins; and maintained at 2% B for 12 mins. Subsequently, the column was re-equilibrated for 5 mins before repeating the cycle. The Q Exactive™ HF mass spectrometer was operated in both positive and negative polarity modes with a spray voltage of 3.5 kV. The capillary temperature was maintained at 320 °C, with a sheath gas flow rate of 35 psi and an auxiliary gas flow rate of 10 L/min. The S-type ionization mirror RF level was set to 60, and the auxiliary gas heater temperature was maintained at 350 °C. Under conditions of 5 kV, the capillary temperature remained at 320 °C, with a sheath gas flow rate of 35 psi, an auxiliary gas flow rate of 10 L/min, an S-type ionization mirror RF level of 60, and an auxiliary gas heater temperature of 350 °C. Metabolite identification was conducted using the KEGG and LIPIDMaps databases. Data acquisition was performed in both positive and negative ion modes; however, statistical analyses were exclusively based on data obtained from the positive ion mode.

#### 2.2.3 Integrated correlation analysis

The associations between significantly altered bacterial genera (identified by 16S rDNA sequencing) and differential metabolites (identified by untargeted metabolomics) were investigated using Pearson correlation analysis.

### 2.3 Statistical analysis

A one-way analysis of variance (ANOVA) was conducted using IBM SPSS Statistics 23.0 (IBM Corporation, Armonk, New York, USA) to assess significant differences among treatment groups. Results are expressed as mean ± standard error of the mean (SEM), with a significance probability *P* < 0.05 considered indicative of a significant difference. Non-targeted metabolomic data were processed using the Linux operating system (CentOS version 6.6; Red Hat, Inc., Raleigh, North Carolina, USA) and R Language (R Core Team, Vienna, Austria), Python Language (Python Software Foundation, Wilmington, Delaware, USA). Data visualization was performed using GraphPad Prism 10.1 (GraphPad Software, San Diego, CA, USA). The correlations between differentially expressed microbial communities and differentially expressed metabolites were assessed using the Pearson correlation coefficient. These tests were conducted as two-sided tests and the raw *P*-values were adjusted for multiple comparisons using the Benjamini-Hochberg method. Correlations with |*r*| > 0.5 and a corrected *P*-value < 0.05 were considered statistically significant.

## 3 Results

### 3.1 The effect of MOE and BZ on the composition and diversity of the microbial community in the cecum of broiler chickens

**Figure 1:**
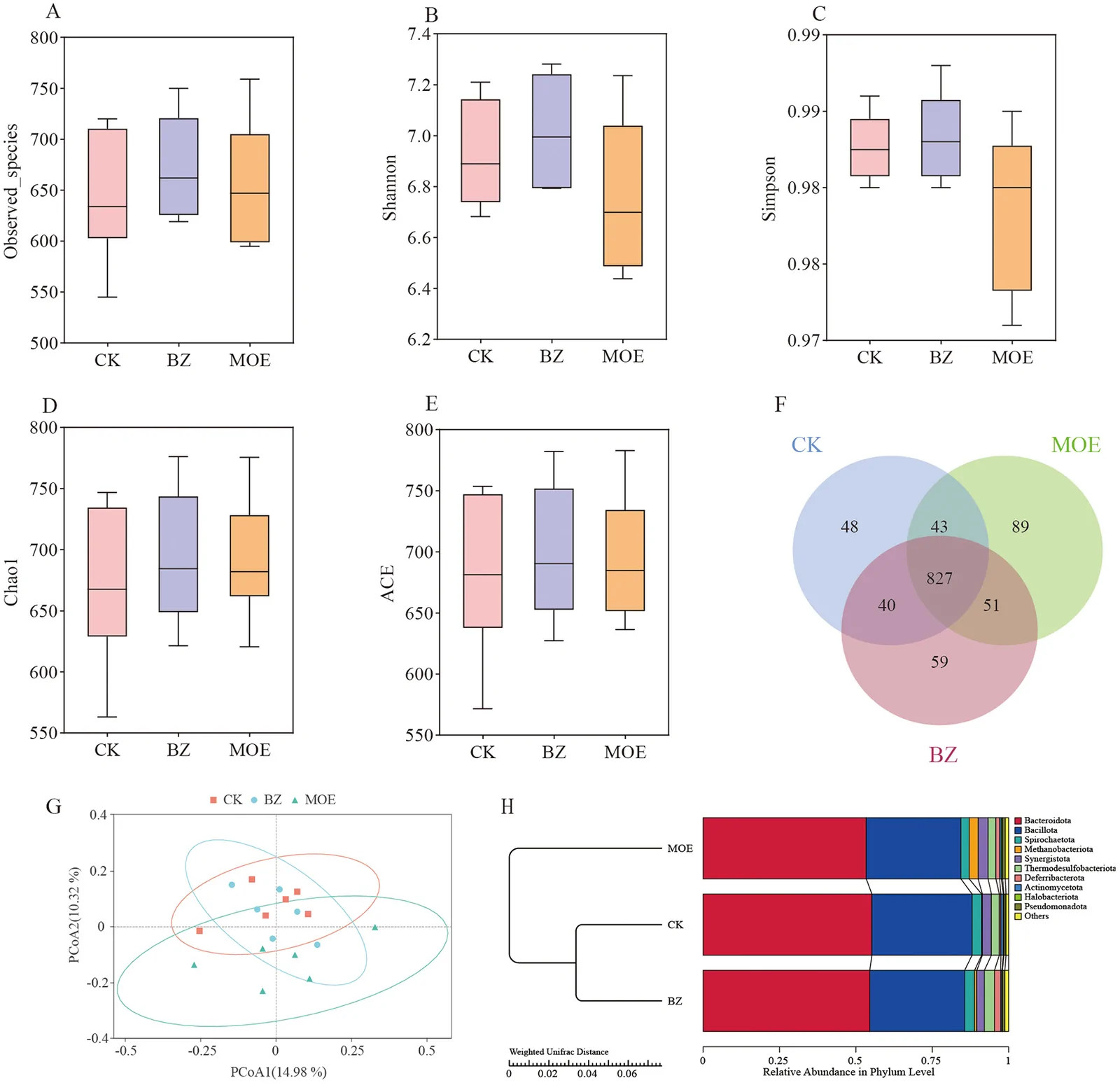
Effects of BZ and MOE on gut microbial diversity in broiler chickens. **(A–E)** α-diversity indices; **(A)** Observed_species; **(B)** Shannon index; **(C)** Simpson's index; **(D)** Chao-1 index; **(E)** ACE index; **(F)** OTU classification plot; **(G)** PCoA analysis based on Bray-Curtis distance. PERMANOVA (Adonis): *R*^2^ = 0.1671, *P* = 0.005; **(H)** UPGMA clustering analysis plot based on the Weighted Unifrac distance matrix. CK, control group; BZ, bacitracin zinc group; MOE, *Moringa oleifera* ethanol extract group.

To investigate the effects of supplementing MOE and BZ on changes in the composition and community structure of the broiler cecal microbiota, we performed 16S rDNA sequencing for microbiome analysis. The results showed that there were no significant differences in Observed_species, Shannon index, Simpson index, Chao1 index, and ACE index among the groups (*P* > 0.05; Figures 1A–E). The Venn diagram revealed that the three groups shared 827 operational taxonomic units (OTUs), with 48 unique to the CK group, 59 unique to the BZ group, and 89 unique to the MOE group (Figure 1F). The PERMANOVA (Adonis) test based on the Bray-Curtis distance matrix indicated that there were significant differences in microbial community structure among the three groups (*R*^2^ = 0.1671, *P* = 0.005); the grouping factor explained 16.71% of the total variation. Although the effect size was in the lower-middle range, the differences between groups were indeed present (Figure 1G). UPGMA cluster analysis further demonstrated that the MOE group exhibited a significant alteration in microbial community structure, while the community structures of the CK and BZ groups were relatively more similar (Figure 1H).

#### 3.1.1 Alterations in the cecal microbial community of broilers supplemented with BZ and MOE

**Figure 2:**
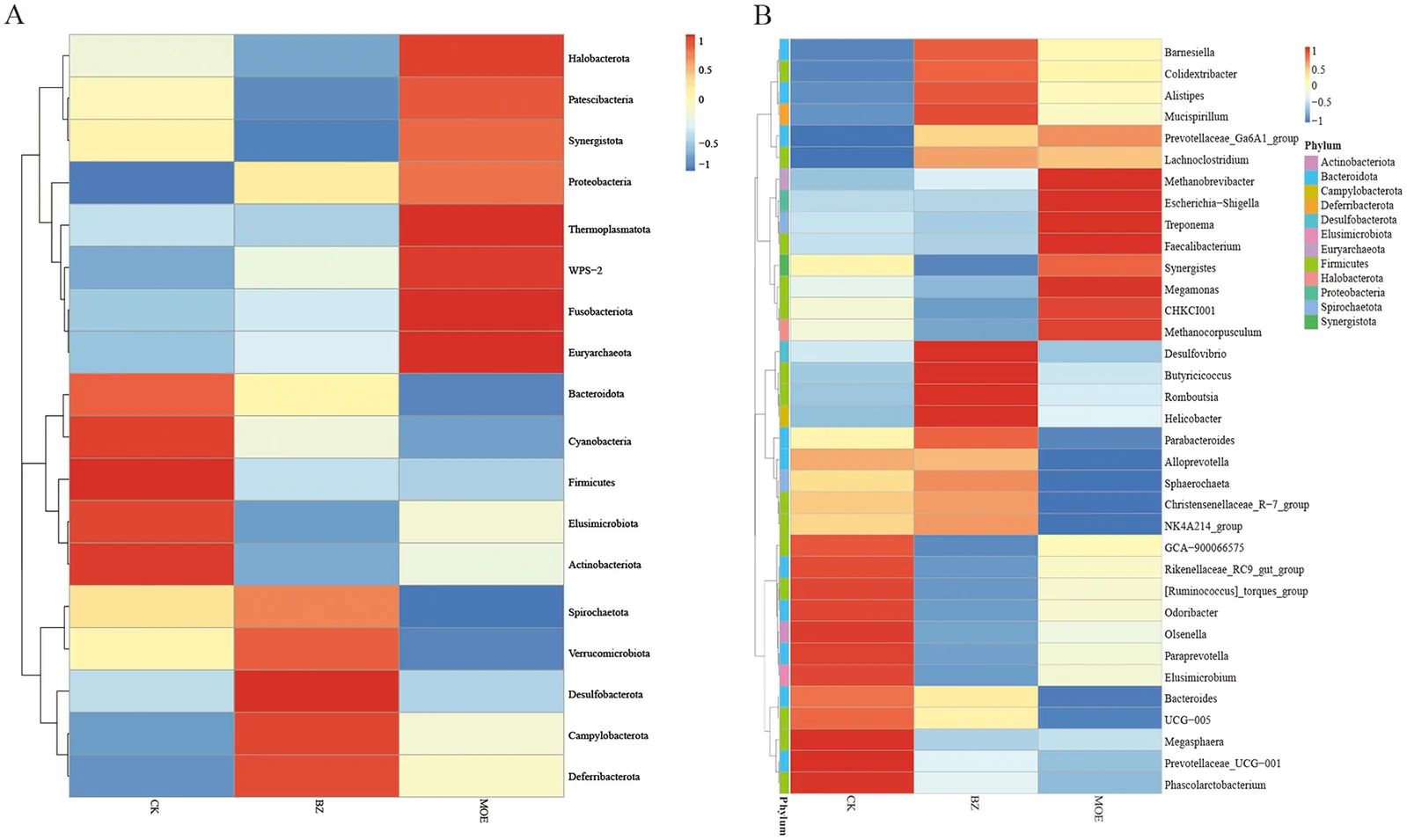
Effects of BZ and MOE on the gut microbiota of broiler chickens. Heatmaps depicting the relative abundance of the top 35 phyla **(A)** and genera **(B)** in the cecal microbiota of broiler chickens (*n* = 6 per group). The color scale represents normalized read abundance, with red indicating higher abundance and blue indicating lower abundance. CK, control group; BZ, bacitracin zinc group; MOE, *Moringa oleifera* ethanol extract group.

Figure 2A shows the heatmap of the top 35 phyla by abundance. Within the CK group, phyla such as *Actinobacteriota, Elusimicrobiota, Firmicutes, Cyanobacteria*, and *Bacteroidota* were notably prevalent. Conversely, the BZ group exhibited a higher abundance of *Deferribacterota, Campylobacterota, Desulfobacterota, Verrucomicrobiota*, and *Spirochaetota*. In the MOE group, there was an increased presence of *Euryarchaeota, Fusobacteriota*, WPS-2, *Thermoplasmatota, Proteobacteria, Synergistota, Patescibacteria*, and *Halobacterota*. These findings indicate that the addition of BZ and MOE significantly altered the gut microbiota composition of broiler chickens. Specifically, pathogenic phyla such as *Campylobacterota, Desulfobacterota*, and *Spirochaetota* were more abundant in the BZ group, while *Fusobacteriota* and *Proteobacteria* showed moderate increases in both the BZ and MOE groups. Overall, both supplements reshaped the microbial community, with BZ promoting a higher abundance of pathogenic phyla. The genus-level clustering heatmap is presented in Figure 2B. Regarding potentially harmful genera, *[Ruminococcus]_torques_group* was more abundant in the CK group, whereas *Helicobacter* and *Desulfovibrio* were enriched in the BZ group, and *Escherichia-Shigella* showed higher abundance in the MOE group. In terms of beneficial or commensal genera, compared with the CK group, MOE supplementation increased the abundance of *Faecalibacterium, Lachnoclostridium, Alistipes*, and *Barnesiella*, while BZ supplementation enhanced the abundance of *Barnesiella, Alistipes, Lachnoclostridium*, and *Butyricicoccus*.

#### 3.1.2 Differential microbial biomarkers identified by LEfSe

**Figure 3:**
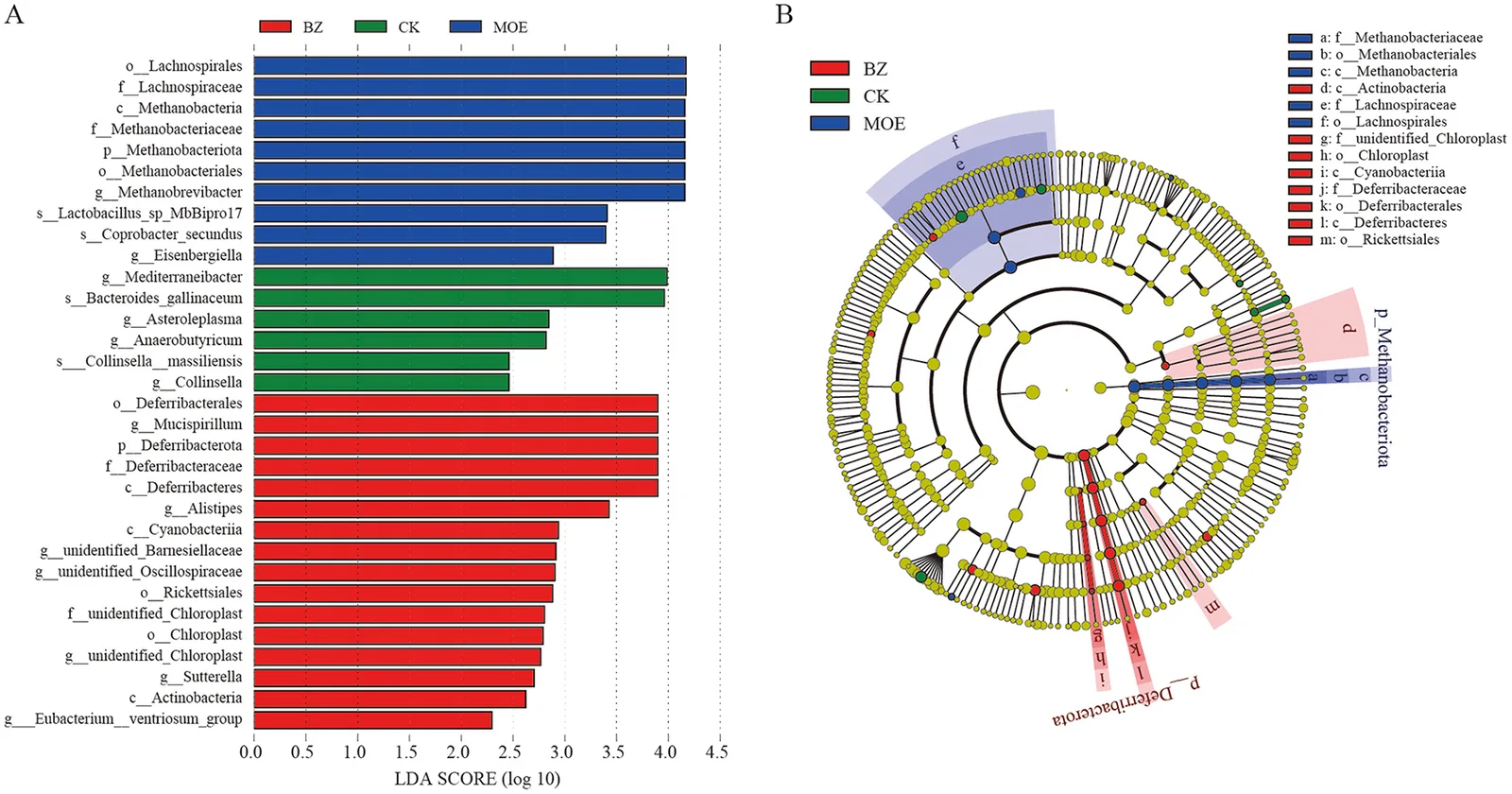
LEfSe analysis of differentially abundant microbial taxa among treatment groups (LDA score > 2). **(A)** LDA value distribution histogram (taxa with LDA score > 2); **(B)** Phylogenetic tree. CK, control group; BZ, bacitracin zinc group; MOE, *Moringa oleifera* ethanol extract group.

The LDA histogram (LDA score > 2; Figure 3A) shows that the differentially expressed microbial communities in the MOE group were predominantly enriched in the *Lachnospirales, Lachnospiraceae, Methanobacteria, Methanobacteriaceae, Methanobacteriales, Methanobrevibacter*, and *Methanobacteriota*. In the BZ group, the differentially expressed microbial communities were primarily enriched in the *Deferribacterales, Mucispirillum, Deferribacterota, Deferribacteraceae*, and *Deferribacteres*. The phylogenetic tree (Figure 3B) further reveals that the differentially expressed microbial communities in the MOE group are primarily distributed within the *Mucispirillales* order under the *Methanobacteria* and *Fusobacteria* classes, whereas those in the BZ group are mainly distributed within the *Deferribacterales* order. These findings indicate that the two groups reshape the gut microbiome through distinct microbial lineages.

### 3.2 The effects of MOE and BZ on the metabolism of the cecal in broiler chickens

**Figure 4:**
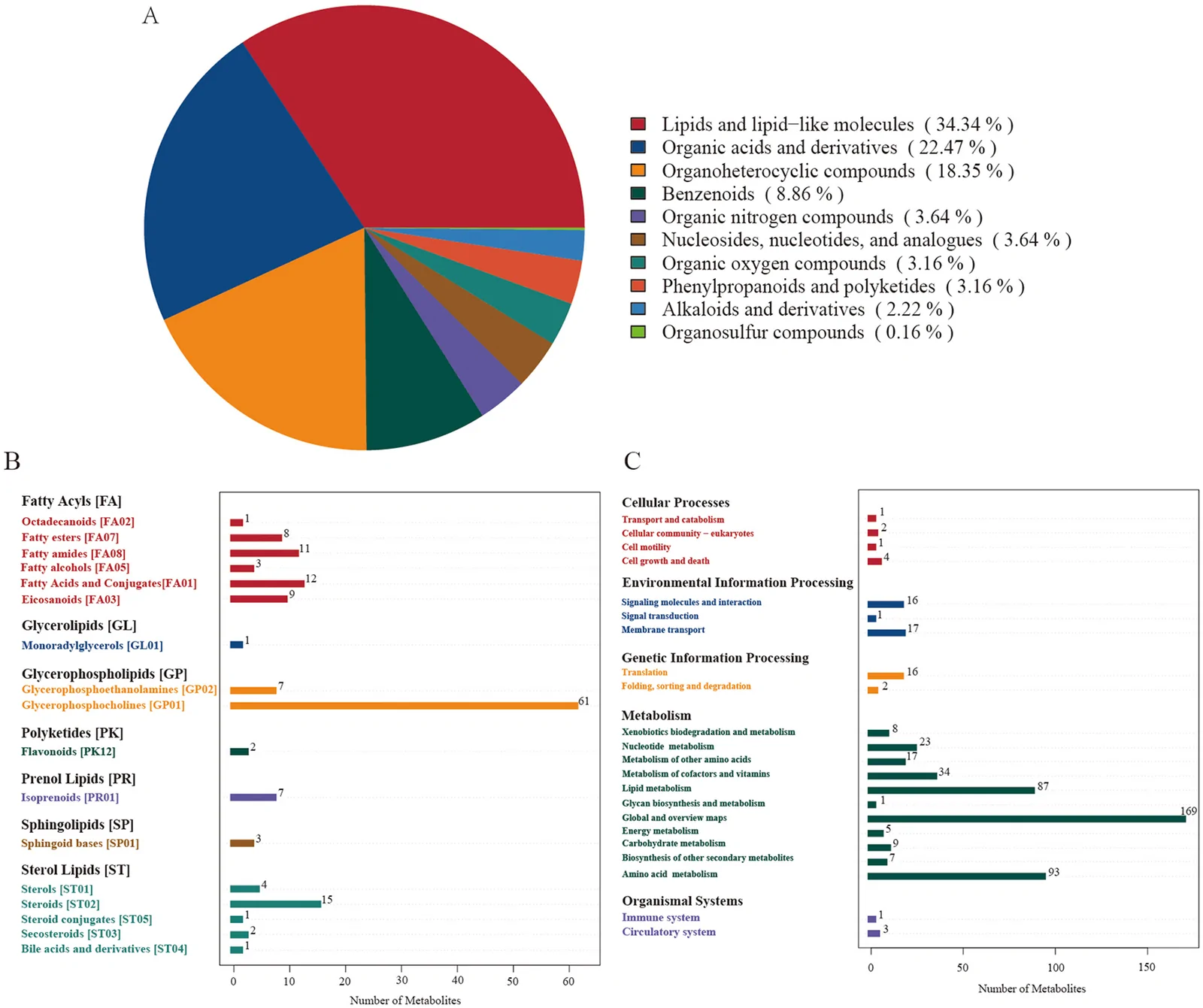
Overview of metabolite classification and pathway annotation in cecal contents. **(A)** Classification of metabolites; **(B)** KEGG pathway annotation; **(C)** Lipidmaps annotation. CK, control group; BZ, bacitracin zinc group; MOE, *Moringa oleifera* ethanol extract group.

To further explore the effects of MOE and BZ supplementation on alterations in broiler cecal metabolites, a metabolomic analysis of the broiler cecal contents was conducted (Figure 4). Classification of the metabolites shows that the major compound category in the samples is lipids and lipid-like molecules, accounting for 34.34% of the total. This is followed by organic acids and derivatives, accounting for 22.47%. The remaining categories were organic oxygen compounds (8.53%), organoheterocyclic compounds (18.35%), and benzenoids (8.86%). Additionally, organic nitrogen compounds, nucleosides, nucleotides and analogs were each detected at 3.64%; organic oxygen compounds, phenylpropanoids, and polyketides at 3.16%; alkaloids and derivatives at 2.22%; and organosulfur compounds at 0.16% (Figure 4A). Pathway analysis was conducted to identify the primary biochemical metabolic and signal transduction pathways in which the metabolites are involved; the KEGG pathway annotation results are shown in Figure 4B: the metabolites were primarily involved in pathways related to cellular processes, environmental information processing, genetic information processing, metabolism, and organismal systems. The annotation results from the LIPID MAPS database are shown in Figure 4C: several major lipid categories were identified, with glycerophospholipids being the most abundant in terms of metabolite count, followed by fatty acyls. In addition, sterol lipids, glycerolipids, polyketides, prenol lipids, and sphingolipids were also detected. Lipid metabolism in this sample is dominated by glycerophospholipids, fatty acyls and sterol lipids, and exhibits a relatively diverse lipid molecular profile.

#### 3.2.1 Serum metabolomic alterations induced by MOE and BZ

**Figure 5:**
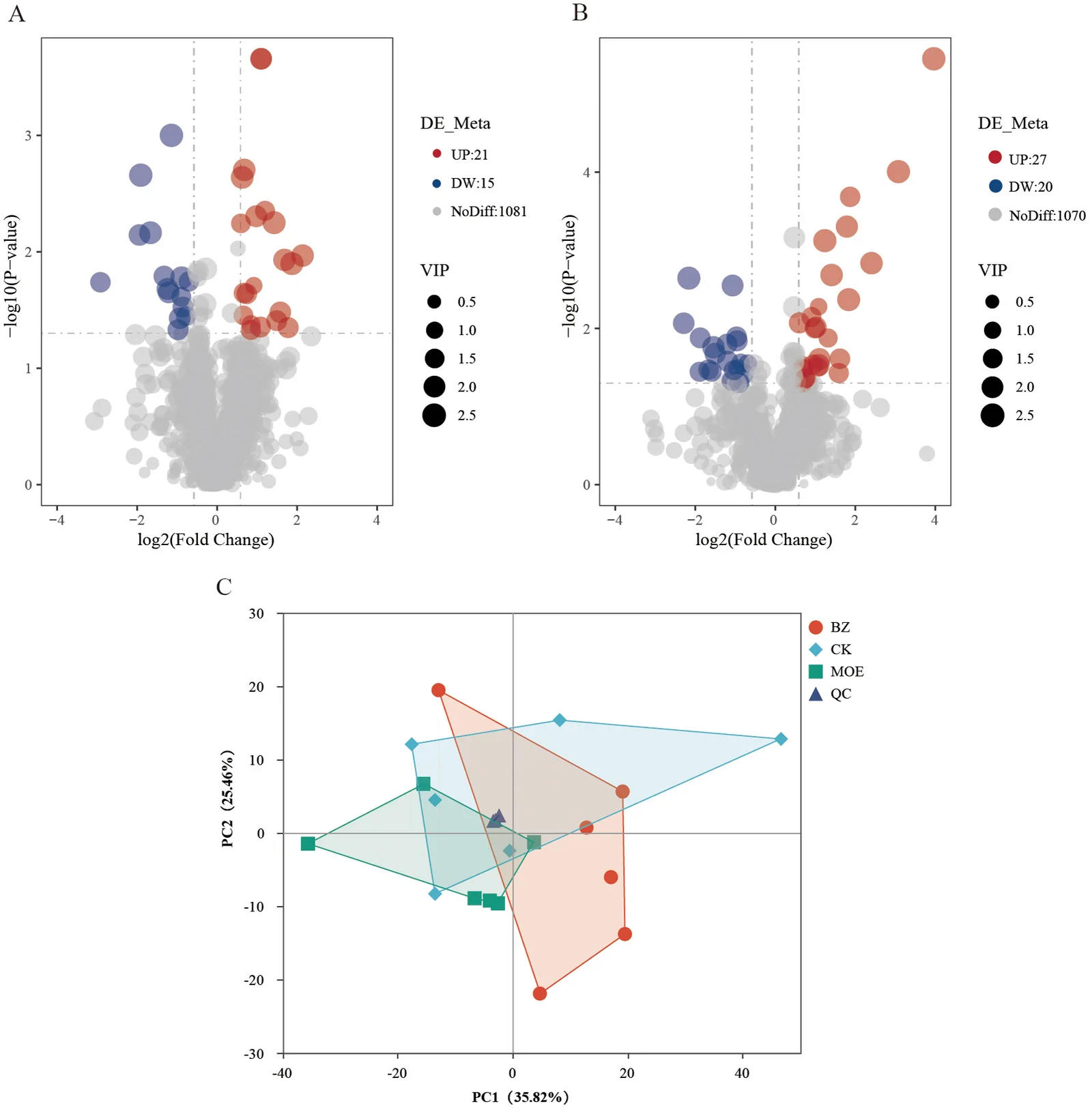
Differential metabolite analysis between treatment groups. **(A)** Volcano plot of differentially expressed metabolites in the BZ vs. CK comparison; **(B)** Volcano plot of differentially expressed metabolites in the MOE vs CK comparison; **(C)** PCA analysis of the total sample. CK, control group; BZ, bacitracin zinc group; MOE, *Moringa oleifera* ethanol extract group.

The volcano plot of differentially expressed metabolites shows that a total of 36 metabolites were identified as significantly different between the BZ and CK groups, comprising 21 that were up-regulated and 15 that were down-regulated. The remaining 1,081 metabolites showed no significant difference between the two groups (*P* > 0.05; Figure 5A). A total of 47 metabolites were identified as significantly different between the MOE and CK groups, comprising 27 that were up-regulated and 20 that were down-regulated. The remaining 1,070 metabolites showed no significant difference between the two groups (*P* > 0.05; Figure 5B). PCA analysis was performed on the peaks extracted from all experimental and QC samples. The results showed that BZ, CK, and MOE were significantly separated, suggesting that MOE differed markedly from the other two groups in terms of the primary sources of variation. Furthermore, samples within each group were relatively clustered, whilst the distinction between groups was clear, indicating that the grouping possessed good biological reproducibility and inter-group heterogeneity (Figure 5C).

#### 3.2.2 KEGG pathway enrichment analysis of differential metabolites

**Figure 6:**
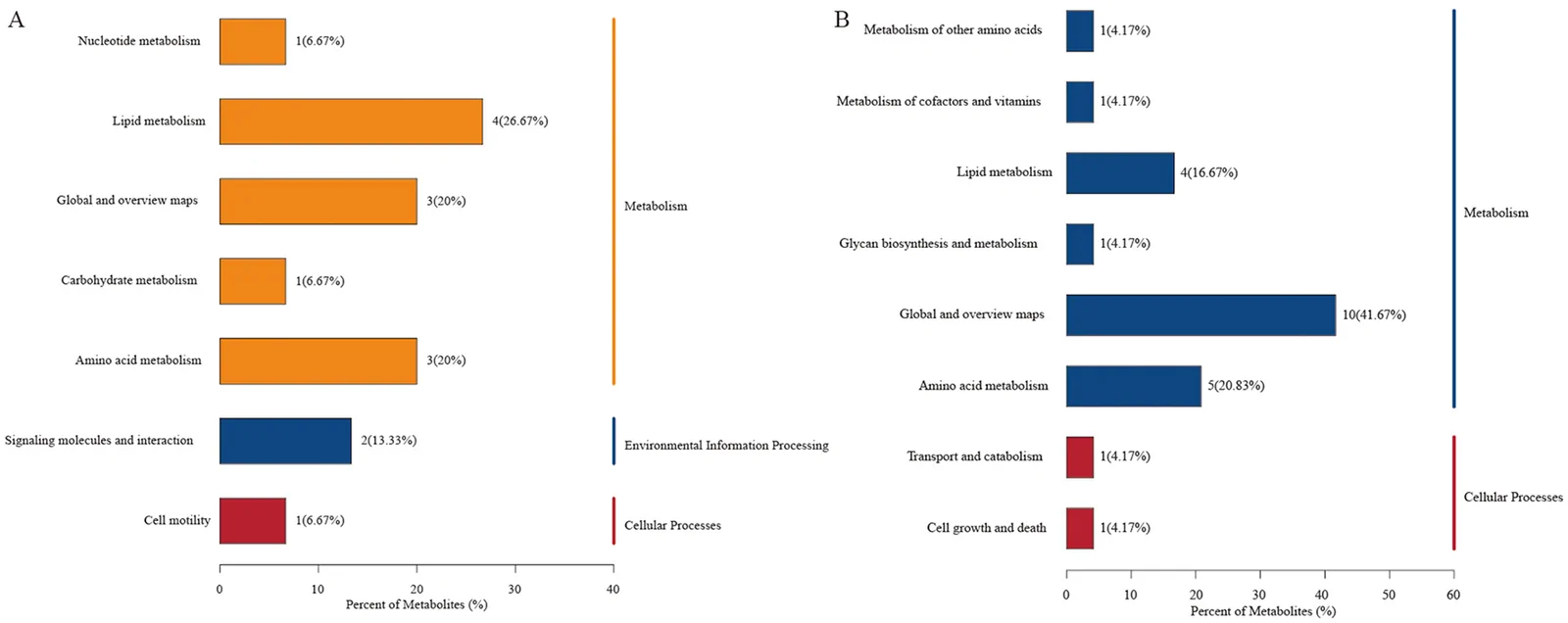
KEGG pathway enrichment analysis of differential metabolites. **(A)** KEGG classification of significantly enriched pathways in the BZ vs. CK comparison enriched; **(B)** KEGG classification of significantly enriched pathways in the MOE vs. CK comparison. CK, control group; BZ, bacitracin zinc group; MOE, *Moringa oleifera* ethanol extract group.

The KEGG classification diagram shows that, compared with the CK group, a total of 15 significantly enriched pathways were identified in the BZ group (*P* < 0.05, FDR < 0.05; Figure 6A), with lipid metabolism accounting for the highest proportion (26.67%), followed by Global and overview maps (20%) and Amino acid metabolism (20%). In addition, Signaling molecules and interactions, Nucleotide metabolism and Carbohydrate metabolism were also represented. BZ treatment primarily induced significant alterations in the lipid, amino acid and global metabolic networks. A total of 24 significantly enriched pathways (*P* < 0.05, FDR < 0.05) were identified in the MOE and CK groups (Figure 6B). Global and overview maps accounted for the highest proportion (41.67%), followed by amino acid metabolism (20.83%) and lipid metabolism (16.67%). Furthermore, pathways such as “Metabolism of other amino acids,” “Metabolism of cofactors and vitamins,” “Glycan biosynthesis and metabolism,” “Transport and catabolism,” and “Cell growth and death” were also enriched. The MOE treatment may primarily influence biological functions by regulating the global metabolic profile and pathways related to amino acid and lipid metabolism.

#### 3.2.3 Hierarchical cluster analysis of differential metabolites

**Figure 7:**
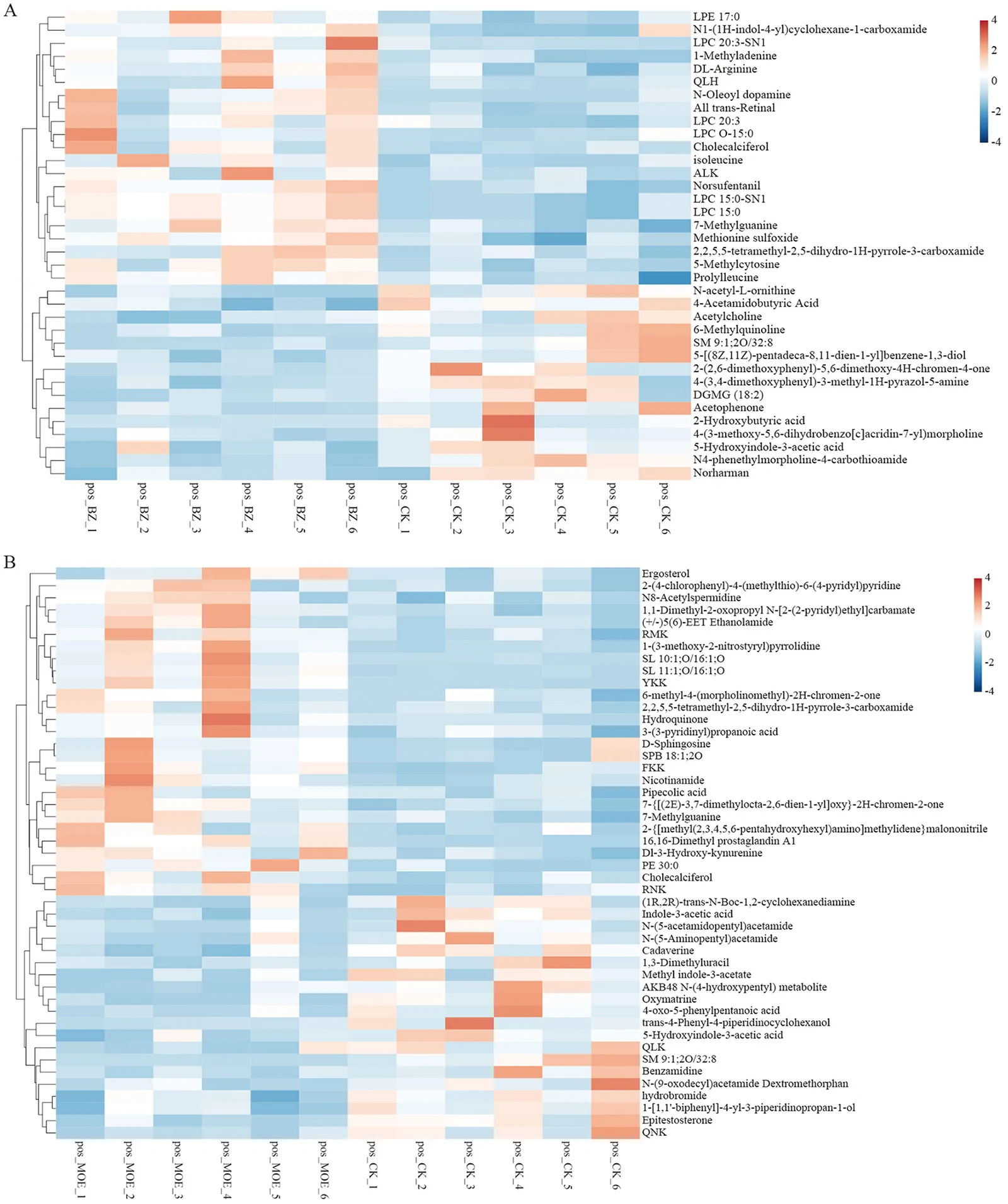
Hierarchical clustering analysis of differentially expressed metabolites between treatment groups. **(A)** Heatmap of metabolites significantly altered in the BZ vs. CK comparison; **(B)** Heatmap of metabolites significantly altered in the MOE vs. CK comparison. CK, control group; BZ, bacitracin zinc group; MOE, *Moringa oleifera* ethanol extract group.

Hierarchical cluster analysis was conducted on the two sets of differentially expressed metabolites to identify differences in metabolic expression patterns between and within the two groups for each comparison pair. The results of the heatmap clustering demonstrated a clear separation in metabolic profiles between the BZ group and the CK group, indicating systematic metabolic differences (Figure 7A). In the CK group, metabolites associated with arginine metabolism, phenolic and flavonoid metabolites, antioxidant-related metabolites, and organic acid/small molecule metabolites were significantly up-regulated, whereas these metabolites exhibited a down-regulated trend following BZ treatment. This observation suggests that the addition of BZ may have inhibited the arginine metabolic pathway, reduced antioxidant capacity, and been accompanied by alterations in the methylation metabolic state. In the BZ group, lipid metabolites were predominant, primarily comprising the lysosomal phosphatidylcholine (LPC) series, lipid signaling molecules, retinoids, and metabolites associated with methylation modifications. This suggests that BZ treatment significantly activated membrane lipid remodeling and oxidative stress-related metabolic pathways. Overall, BZ treatment exhibited significant metabolic differences compared to CK, primarily manifested as downregulation of arginine metabolism and antioxidant capacity, as well as reprogramming of lipid metabolism. In contrast, MOE treatment significantly altered the expression levels of various metabolites; in terms of lipid metabolism, several sphingolipids were significantly upregulated in the MOE group, while multiple metabolites in the tryptophan metabolic pathway were significantly downregulated. Additionally, the expression of polyamine metabolites, cholecalciferol, and nicotinamide was also altered in the MOE-treated group (Figure 7B). This indicates that MOE treatment significantly altered the expression levels of multiple metabolites, involving various biological processes such as lipid metabolism, amino acid metabolism, polyamine metabolism, and vitamin metabolism.

### 3.3 Correlation analysis

**Figure 8:**
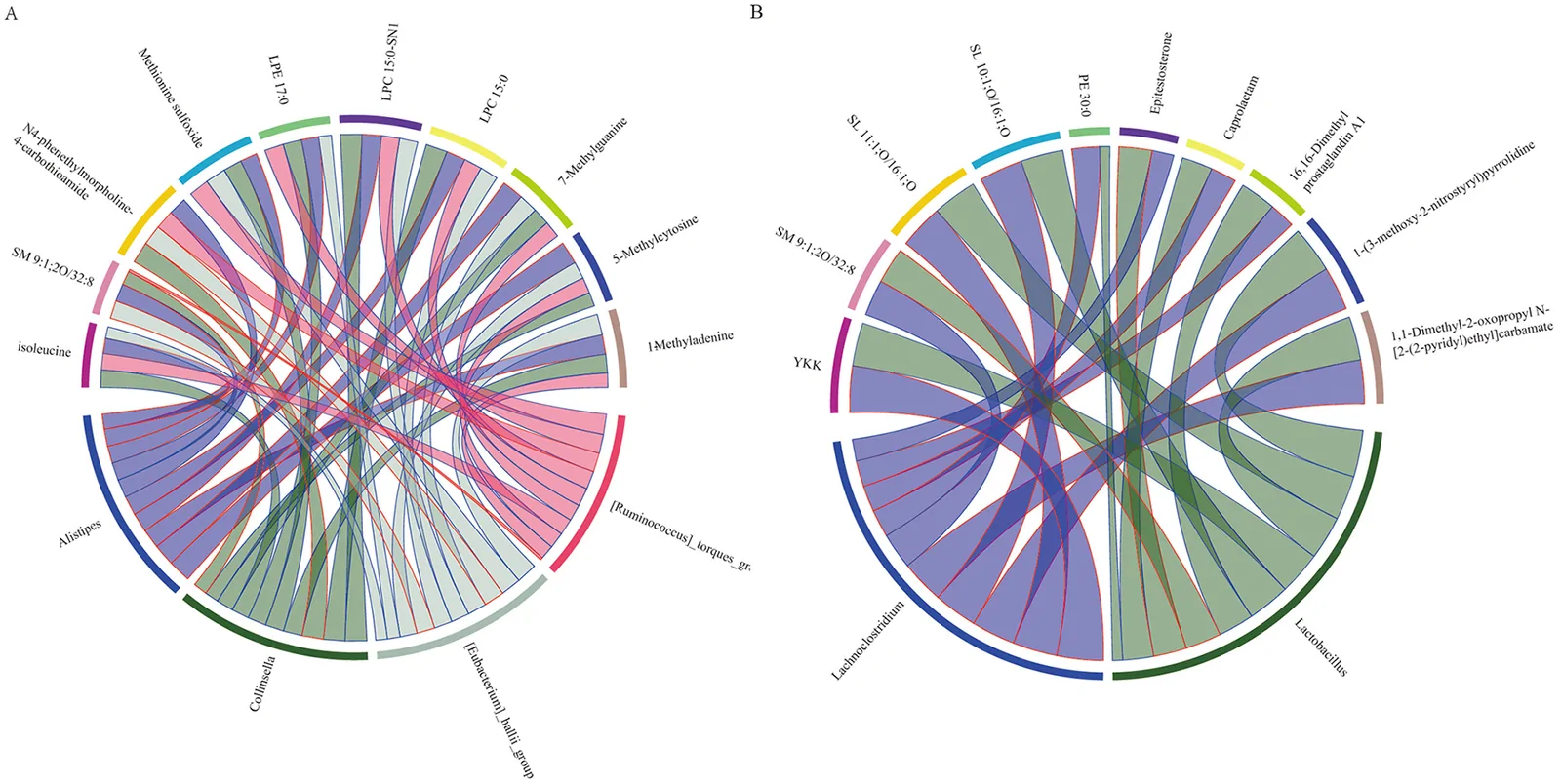
Correlation analysis. Chord diagrams showing significant correlations (Pearson, |*r*| > 0.5, *P* < 0.05) between differentially abundant bacterial genera and metabolites in the BZ vs. CK group **(A)** and MOE vs. CK group **(B)**. Red links indicate positive correlations; blue links indicate negative correlations. Line width is proportional to the correlation coefficient (*r*).” CK, control group; BZ, bacitracin zinc group; MOE, *Moringa oleifera* ethanol extract group.

Figure 8A shows that, based on Pearson correlation analysis, 27 significantly correlated bacterial genus–metabolite pairs (|*r*| > 0.5, *P* < 0.05) were identified in the BZ group. These pairs involved four differentially expressed bacterial genera (*Alistipes, Collinsella, Eubacterium hallii* group, and *Ruminococcus torques* group) and 10 differentially expressed metabolites, primarily methylated metabolites (1-methyladenine, 5-methylcytosine, and 7-methylguanine), haemolytic phosphatidylcholine (LPC 15:0 and LPC 15:0-SN1) and haemolytic phosphatidylethanolamine (LPE 17:0). Of these, Alistipes showed a significant positive correlation with 1-methyladenine, 5-methylcytosine, and 7-methylguanineand LPC 15:0. In contrast, *Collinsella* and the *Eubacterium hallii* group showed significant negative correlations with these metabolites (|*r*| > 0.58, *P* < 0.05). This suggests that BZ treatment leads to the development of an “alkylation metabolite–lipid metabolite” network centered on Alistipes. In the MOE group (Figure 8B), a total of 12 significantly correlated genus–metabolite pairs were identified by Pearson correlation analysis (|*r*| > 0.5, *P* < 0.05), primarily involving the two genera *Lachnoclostridium* and *Lactobacillus*. *Lachnoclostridium* was significantly correlated with YKK, SL 11:1;O/16:1;O, 1-(3-methoxy-2-nitrostyryl) pyrrolidine, SL 10:1;O/16:1;O and 1,1-dimethyl-2-oxopropyl N-[2-(2-pyridyl)ethyl]carbamate, and negatively correlated with SM 9:1;2O/32:8. Interestingly, there were negative correlations between *Lachnoclostridium* and *Lactobacillus* and most lipid-related metabolites (SL series, SM and YKK). This suggests that these two genera may play a regulatory role in host sphingolipid metabolism under MOE intervention. These findings indicate that specific metabolites exhibit differential associations with the gut microbiota depending on the dietary intervention.

## 4 Discussion

### 4.1 Differential modulation of cecal microbiota: MOE promotes beneficial symbionts whereas BZ alters pathogenic and protective genera

The gut harbors a complex ecosystem comprising a diverse array of microorganisms, which play a crucial role in the processing of nutrients and exert a profound influence on the host's immune system (Iliev et al., 2025). The composition of the gut microbiota in broiler chickens is closely linked to nutrient digestion and absorption, immune status and intestinal barrier function, and is also influenced by diet type (Ma et al., 2022; Munyaka et al., 2016). Previous studies have found that phytochemicals in moringa, such as flavonoids, alkaloids and saponins, possess antimicrobial properties capable of inhibiting pathogenic bacteria such as *Escherichia coli* and *Salmonella*, whilst promoting the growth of beneficial bacteria (Nantapo, 2018). Research by Wizrah et al. (2025) found that moringa leaf extract exhibits antibacterial activity against Gram-negative bacteria, effectively inhibiting the growth of *E. coli, Sh. shiga, P. aeruginosa*, and *Sh. sonnei*. Furthermore, studies have indicated that the addition of up to 5% moringa leaf powder to feed can have a positive impact on broiler chickens by improving antioxidant status, meat quality, nutrient digestibility, and carcass characteristics, as well as preventing intestinal pathogens, without causing any adverse effects (El-Tazi, 2014; Jiang et al., 2023; Mahfuz and Piao, 2019; Soundararajan et al., 2023). Furthermore, evidence suggests that supplementation with *Moringa oleifera* can influence poultry product quality and the gut microbial ecosystem (Akib et al., 2024). In our study, we observed that the cecal microbiota of broiler chickens supplemented with MOE exhibited a significant enrichment of Euryarchaeota at the phylum level, with its relative abundance approximately four-fold higher than that of the CK group. LEfSe analysis further identified *Lachnospiraceae* and *Methanobacteriaceae* as key differential biomarkers in the MOE group. *Lachnospiraceae* are important butyrate-producing bacterial groups (Apajalahti and Vienola, 2016); their metabolite, butyrate, serves as the primary energy source for colonic cells, providing energy to colonic epithelial cells and helping to prevent colonic inflammation (Canani et al., 2011). It is also capable of inhibiting the proliferation of pathogenic and harmful bacteria in the gut, whilst promoting the colonization and development of beneficial bacteria (Devillard et al., 2007). At the genus level, the abundance of *Methanobrevibacter* was significantly elevated in the MOE group, whilst beneficial genera such as *Faecalibacterium* and *Lachnoclostridium* also showed a trend toward enrichment. This is consistent with the findings of Zhang et al. (2023), who by substituting rapeseed meal with *Moringa oleifera* leaf powder (MOLP) at different concentrations, discovered that 5% MOLP promotes the colonization of *Faecalibacterium* in the broiler chicken gut. *Faecalibacterium* comprises anaerobic bacteria capable of producing butyrate. It has proven probiotic properties and is regarded as a marker of gut health (Stanley et al., 2016), with a negative correlation to inflammatory bowel disease and colorectal cancer (Cao et al., 2014). They play a crucial role in providing energy to colonic cells, producing anti-inflammatory compounds that are beneficial to gut health, such as butyrate and salicylic acid (Sokol et al., 2008), and regulating lipid metabolism and blood glucose homeostasis in broiler chickens (Yang et al., 2018). It also improves production efficiency by enhancing feed conversion and reducing feed intake (Lacey et al., 2016). A reduction in its numbers is closely associated with inflammatory bowel disease and irritable bowel syndrome (Qiu et al., 2013). *Lachnoclostridium* is capable of producing a variety of beneficial metabolites (such as lactic acid, short-chain fatty acids and bacteriocins); these antimicrobial metabolites can inhibit invading pathogens (Wan et al., 2024) and alleviate intestinal inflammation (Shang et al., 2020), suggesting that MOE may improve the intestinal microbiota by promoting synergistic interactions among various probiotic strains. Research by Abbas et al. (2024) demonstrated that following 14 consecutive days of administration of a broad-spectrum antibiotic mixture in the drinking water of broiler chicks, the relative abundance of the *Firmicutes* phylum, *Lactobacillus*, and *Bacillus* in the broiler cecum decreased significantly, whilst the relative abundance of the Bacteria order, *Proteobacteria* phylum, *Cyanobacteria* phylum, and *Enterococcus* increased significantly. In this study, the proportion of potentially pathogenic phyla such as *Desulfobacterota, Deferribacterota*, and *Proteobacteria* was significantly increased in the BZ group at the phylum level. LEfSe analysis identified the *Deferribacterales* and the *Mucispirillum* as differential biomarkers for the BZ group. *Deferribacterota* enrichment has previously been observed in antibiotic-induced dysbiosis (Behr et al., 2018; Wen et al., 2017), while *Mucispirillum* has been reported to be significantly associated with antibiotic residues (Liang et al., 2024). These findings corroborate results showing enrichment of Deferribacterota and Desulfobacterota at the phylum level in the BZ group, and further validate the trend that BZ treatment promotes the relative expansion of specific anaerobic bacterial communities at the genus/order level. Proteobacteria are putrefactive bacteria that can disrupt gastrointestinal function and trigger various diseases, such as metabolic disorders and inflammatory bowel disease (Yu et al., 2022; Zeng et al., 2017). However, at the genus level, the BZ group also exhibited enrichment of beneficial bacterial genera such as *Barnesiella, Alistipes*, and *Butyricicoccus*. Among these, *Butyricicoccus* is a butyrate producer that has demonstrated good anti-inflammatory effects in human and animal studies (Geirnaert et al., 2014); it is crucial for maintaining the activity of mucosal immune cells, the integrity of the intestinal epithelium, barrier function, and the suppression of the growth of pathogenic bacteria (Liu et al., 2021). *Alistipes*, a propionic acid-producing bacterium, is negatively correlated with pro-inflammatory cytokines in broiler chickens (Li et al., 2022; Polansky et al., 2016) and is also associated with necrotic enteritis and infection by the pathogen *Emeryella tenella* (Song et al., 2023; Yu et al., 2023). *Barnesiella* has been shown to inhibit the growth of harmful microbiota and maintain the balance of the intestinal microbiome (Guo et al., 2023). This indicates that the effect of BZ treatment on microbiota structure is bidirectional.

### 4.2 Integrated metabolomic and microbiomic analyses reveal MOE and BZ specific metabolic association pattern

Metabolomics results further support these findings. The MOE group significantly influenced the sphingolipid, tryptophan and polyamine metabolic pathways. With regard to lipid metabolism, sphingolipids such as D-sphingosine and SPB 18:1;2O were significantly up-regulated in the MOE group. Sphingolipids and their metabolites (such as ceramides and sphingosine-1-phosphate) are not only important structural components of cell membranes but also play a crucial role in cell signaling, inflammation regulation and the regulation of intracellular transport (Hannun and Obeid, 2008). With regard to amino acid metabolism, several metabolites in the tryptophan metabolic pathway (such as DI-3-hydroxy-kynurenine, indole-3-acetic acid, and 5-hydroxyindole-3-acetic acid) exhibited abnormal expression in the MOE group. Tryptophan metabolites are not only involved in neurotransmitter synthesis (e.g., serotonin) but are also closely associated with immune tolerance and oxidative stress (Cervenka et al., 2017). Among these, activation of the kynurenine pathway has been shown to be associated with inflammatory diseases and neurodegenerative disorders (Pegg, 2016; Schwarcz and Stone, 2017). Therefore, MOE may influence the function of the immuno-neural axis by regulating tryptophan metabolism, thereby exerting a modulatory effect on the host's immune status. Furthermore, changes in polyamine metabolites (such as N8-acetylspermidine and cadaverine) suggest that MOE may influence cell proliferation and autophagy processes. Polyamines play a crucial role in cell growth, differentiation and stress responses, and their metabolic dysregulation is frequently associated with pathological conditions such as tumors and aging (Pegg, 2016). It is worth noting that the expression of vitamin D3 (cholecalciferol) and nicotinamide was also altered in the MOE group. Vitamin D not only regulates calcium and phosphorus metabolism but also possesses immunomodulatory functions (Bouillon et al., 2019); nicotinamide, as a precursor of NAD^+^, plays a vital role in energy metabolism, DNA repair and anti-aging (Yoshino et al., 2018). These changes further support the broad influence of MOE on cellular metabolic homeostasis. KEGG enrichment analysis revealed that differentially expressed metabolites in the MOE group were primarily enriched in lipid and amino acid metabolic pathways, which aligns closely with the changes in microbial community structure. Correlation analysis further revealed that *Lachnoclostridium* was significantly positively correlated with various lipid metabolites, whilst *Lactobacillus* was associated with steroid hormones and sphingolipid metabolites, suggesting potential interconnections between these genera and specific metabolic pathways that warrant further investigation. These findings suggest that MOE may exert its comprehensive regulatory effect on gut health by synergistically modulating the abundance of butyrate-producing bacteria and methanogenic archaea, thereby influencing multiple host metabolic pathways. The BZ group was primarily characterized by significant activation of lipid metabolism and downregulation of arginine metabolism. The lysosomal phosphatidylcholine (LPC) series was significantly upregulated in the BZ group; LPC, as an important signaling molecule, is involved in inflammatory responses, oxidative stress and cell membrane remodeling (Schmitz and Ruebsaamen, 2010). Concurrently, the antioxidant-related metabolite acetylcysteine and arginine metabolism intermediates N-acetyl-L-ornithine and 4-acetamidobutyric acid were downregulated in the BZ group, suggesting that BZ treatment may have weakened the gut's antioxidant defense capacity. KEGG analysis also confirmed that lipid metabolism and amino acid metabolism were the primary enriched pathways among differentially expressed metabolites in the BZ group. Correlation analysis revealed that *Alistipes* was positively correlated with various methylated metabolites, whilst *Collinsella* was negatively correlated with most metabolites, forming a association pattern centered on these two genera. Overall, BZ treatment, whilst activating lipid metabolism, was accompanied by an increase in the phylum of opportunistic pathogens and a decline in antioxidant capacity; its overall effect may exhibit the complex characteristic of “metabolic activation coexisting with microbiome-related risks.”

In summary, this study used association analysis to create distinct “metabolite-microbe” interaction networks for the BZ and MOE groups. The BZ group centers on the genera *Alistipes* and *Collinsella*, and is associated with methylated metabolites and lipid molecules. In contrast, the MOE group centers on the genera *Lachnoclostridium* and *Lactobacillus*, and is associated with sphingolipids, steroid hormones, and nitrogen-containing compounds. These differences reflect the distinct regulatory patterns of the two additives on the gut microbiota. MOE is associated with the enrichment of probiotic communities and beneficial metabolites, whereas BZ introduces a certain degree of microbial risk while activating specific metabolic pathways. These findings reveal distinct patterns of association between microbes and metabolites in the MOE and BZ groups, providing insights at multiple omics levels into the potential mechanisms underlying the modulatory effects of MOE on gut microbiota and host metabolism. However, it is important to acknowledge that these associations are correlative; thus, future studies employing mechanistic approaches are necessary to establish causal relationships.

## 5 Conclusion

In this study, we employed 16S rRNA gene sequencing and non-targeted metabolomics analysis of broiler cecal contents were conducted to systematically evaluate the effects of BZ and MOE treatments on the structure of the gut microbiota and its metabolites. The results indicated that although BZ and MOE treatments did not significantly alter the overall diversity of the broiler gut microbiota, they induced systemic remodeling at both the microbial composition and metabolic profile levels. MOE was associated with the enrichment of key bacterial groups, such as *Lachnospiraceae* and *Methanobacteriaceae*, and the increased production of beneficial metabolites, including sphingolipids and polyamines. This suggests a coordinated microbial-metabolic response that may contribute to gut health; BZ, however, exhibited a bidirectional effect, activating lipid metabolism while concurrently increasing the abundance of certain pathogenic bacterial phyla and downregulating antioxidant capacity. This study provides multi-omics-level theoretical support for investigating the synergistic regulatory mechanisms of MOE as a feed additive on gut microbiota and host metabolism, and lays a theoretical foundation for the subsequent screening and application of MOE as a functional additive.

## Data Availability

The datasets presented in this study have been deposited in publicly accessible repositories. The 16S rRNA gene sequencing raw data are available in the NCBI Sequence Read Archive (SRA) under BioProject ID PRJNA1474761. The metabolomics raw data have been deposited in the EMBL-EBI MetaboLights repository under accession number MTBLS15049.
